# An Insight into Pathophysiological Features and Therapeutic Advances on Ependymoma

**DOI:** 10.3390/cancers13133221

**Published:** 2021-06-28

**Authors:** Seung-Hee Seo, Shamrat Kumar Paul, Mita Shikder, Mushira Khanam, Popy Ghosh, Tasnin Al Hasib, Kazi Ahsan Ahmed, Suranjana Sikdar, Md Jamal Uddin, Youngjoo Kwon

**Affiliations:** 1 Graduate School of Pharmaceutical Sciences, College of Pharmacy, Ewha Womans University, Seoul 03760, Korea; shseo33@ewhain.net; 2 Department of Biochemistry and Molecular Biology, Life Science Faculty, Bangabandhu Sheikh Mujibur Rahman, Science and Technology University, Gopalganj 8100, Bangladesh; shamratpaul.bmb@gmail.com (S.K.P.); mitashikder02@gmail.com (M.S.); mknm9806@gmail.com (M.K.); gpopy97@gmail.com (P.G.); tasnim22.hasib@hotmail.com (T.A.H.); kaziahsanahmed.bmb@gmail.com (K.A.A.); 3 Bio-Science Research Initiative, Gopalganj 8100, Bangladesh; 4 ABEx Bio-Research Center, East Azampur, Dhaka 1230, Bangladesh; 5 Department of Microbiology, Faculty of Biological Sciences, University of Chittagong, Chittagong 4331, Bangladesh; suranjana.micro@std.cu.ac.bd

**Keywords:** brain tumor, ependymoma, oncogene, tumor diagnosis, tumor treatment

## Abstract

**Simple Summary:**

Although biological information and the molecular classification of ependymoma have been studied, the treatment systems for ependymoma are still insufficient. In addition, because the disease occurs infrequently, it is difficult to obtain sufficient data to conduct large-scale or randomized clinical trials. Therefore, this study is intended to emphasize the importance of understanding its pathological characteristics and prognosis as well as developing treatments for ependymoma through multilateral studies.

**Abstract:**

Glial cells comprise the non-sensory parts of the central nervous system as well as the peripheral nervous system. Glial cells, also known as neuroglia, constitute a significant portion of the mammalian nervous system and can be viewed simply as a matrix of neural cells. Despite being the “Nervenkitt” or “glue of the nerves”, they aptly serve multiple roles, including neuron repair, myelin sheath formation, and cerebrospinal fluid circulation. Ependymal cells are one of four kinds of glial cells that exert distinct functions. Tumorigenesis of a glial cell is termed a glioma, and in the case of an ependymal cell, it is called an ependymoma. Among the various gliomas, an ependymoma in children is one of the more challenging brain tumors to cure. Children are afflicted more severely by ependymal tumors than adults. It has appeared from several surveys that ependymoma comprises approximately six to ten percent of all tumors in children. Presently, the surgical removal of the tumor is considered a standard treatment for ependymomas. It has been conspicuously evident that a combination of irradiation therapy and surgery is much more efficacious in treating ependymomas. The main purpose of this review is to present the importance of both a deep understanding and ongoing research into histopathological features and prognoses of ependymomas to ensure that effective diagnostic methods and treatments can be developed.

## 1. Introduction

Ependymal cells are one of four types of glial cells found in the central nervous system (CNS). Their main function is to secrete, circulate, and maintain the homeostasis of the cerebrospinal fluid (CSF) that fills the ventricles of the CNS [1]. CSF plays many important roles in protecting the brain from shock and supporting nutrient delivery and waste removal [2]. Ependymal cells circulate CSF through the ventricles using motile, hair-like projections called cilia on the cell surface [3]. Aquaporin channels in ependymal cells are responsible for transporting water in both directions between the blood and the ventricles [4]. These functions are essential for the formation of CSF and maintaining CSF homeostasis. When an ependymoma develops, it blocks the flow of CSF and increases intracranial pressure [5]. Thus, an ependymoma presents primary symptoms associated with headache, nausea, or vomiting or the sudden enlargement of an infant’s head size. An ependymoma is a rare form of tumor that usually develops in the cranial ventricular system or spinal cord. It accounts for 2 to 9% of all intracranial tumors and up to 12% of pediatric brain tumors [6,7]. It is a locally aggressive tumor with metastatic potentiality, causing a devastating and dreadful pediatric disease [8]. It involves the three major anatomic compartments of the CNS (supratentorial brain, posterior fossa, and spinal cord), affects both adults and children, and has a higher morbidity rate in children [9,10]. These tumors may form anywhere in the ventricular system and spinal cord of the CNS. However, these tumors may vary depending on the patient’s age and gender and the histological location of onset disease [11]. Based on the ependymoma’s location, about 60% of ependymomas are infratentorial, whereas 40% are supratentorial [12]. About 94% of supratentorial ependymal tumors are larger than 4 cm in size, whereas most infratentorial ependymal tumors are smaller than supratentorial ependymomas [13]. Ependymomas occur more frequently in men than in women, at a ratio of 1.77:1 [11,14,15,16].

According to the guidelines of the WHO, there are three subgroups of ependymoma, indicated as grade I, II, and III. Grade I is easier to detect and treat, especially in adults. Grades II and III are the conventional types, and grade III is more aggressive than those. It shows extensive tissue necrosis as well as metastasis [9,17]. Radial glial cell (RGC) mutation is thought to be the original source of ependymomas [18]. Chromosomal abnormalities also play a significant role in ependymoma development. The dissemination of ependymoma, found in 11.4% of cases [19], affects extraneural organs such as the lungs, liver, and kidneys. Both magnetic resonance imaging (MRI) and computed tomography (CT) are used to diagnose ependymoma [20,21]. MRI is often used as a primary diagnosing procedure for better contrast and image quality [21,22]. In general, chemotherapy, radiotherapy (RT), and surgery have been the most common therapeutic approaches for ependymoma. Surgery and RT are considered as primary therapeutic approaches, while chemotherapy has some limitations [11]. Pediatric patients with ependymoma possess a higher morbidity rate; therefore, the management of ependymoma is a real challenge [16]. Remarkable progress may have been made in the treatment of ependymoma over the past few decades. Treatment plans can vary from country to country, though surgery and radiation are the mainstays [10]. Molecular biomarkers may reveal a new method to distinguish various symptoms according to the grading system. In addition, new information has been discovered by genetic and transcriptomics studies [23]. Hence, clinical management is still difficult. This study attempts to review and summarize the histopathological features, diagnosis, prognosis, and probable efficacious therapeutic measures of ependymoma. A juxtaposition of the morphological features of different classes of ependymoma is also an objective of this review. Scrutinizing current methods of diagnosis and unraveling possible diagnostic methods is one of the prime concerns of the study. This review also aims to summarize the clues and associated limitations and future perspectives on what might be the most promising therapeutic measures for combating ependymoma.

## 2. Methods

The literature of this study has been searched using for Google Scholar and PubMed, and the major keywords were brain tumor, ependymoma, oncogene of ependymoma, ependymoma diagnosis, mechanisms, and ependymoma tumor treatment. We filtered the most recently published papers, and articles related to clinical features were searched using the similar keywords mentioned above.

## 3. Histopathological Classification of Ependymoma

There are several types of brain tumors. According to the 2016 classification by the World Health Organization (WHO), an ependymoma is traditionally classified into three histopathological subtypes, including WHO grade I (myxopapillary ependymoma, subependymoma), WHO grade II (ependymoma), WHO grade III (anaplastic ependymoma), and WHO grade II or III (*RELA* fusion-positive) (Table 1) [24]. A myxopapillary ependymoma, a slow-growing low-grade tumor, accounts for approximately 10% of ependymomas [25]. A myxopapillary ependymoma is a rare tumor located in the conus medullaris, cauda equinea, and filum terminale of the spinal cord [26]. It mainly occurs in young adults with an average of 36 years of age. The histopathological feature is considered to be the arrangement of the tumor cells around the vascularized mucous matrix core in the form of a papilla [17]. In addition, a myxopapillary ependymoma is known to express the glial fibrillary acidic protein (GFAP). It has been found that very high levels of GFAP are expressed in the blood of myxopapillary ependymoma patients with pleural and lung metastases [27]. GFAP is used in clinical neuropathology as a standard immunohistochemical marker to identify glial tumor differentiation and gliosis [28]. A subependymoma grows slowly near the ventricles, which accounts for about 8% of all ependymomas. Subependymoma is found mainly in middle-aged adults rather than in children, although gender and age are not associated with the prognosis of the disease [29]. A subependymoma is an intraventricular tumor that can constrain cerebrospinal fluid (CSF) dynamics [30]. On radiological findings, a subependymoma occurs most frequently in the fourth of the ventricles (50 to 60%) and is also found in the lateral ventricle (30 to 40%), the third ventricular, or central canal of the spinal cord (occasionally) [31,32]. A WHO grade II ependymoma may appear at any age along the ventricles or spine [33]. About 60% of ependymomas occur most often in the fourth ventricle, located on the posterior fossa. About 30% appear primarily in the lateral or third ventricles placed in the supratentorial location, and the remaining 10% are found in the spine. In children, tumors are often found in the posterior fossa, while in adults from 30 to 50 years old, they are usually detected in the vertebral position [33]. An ependymoma comprises the following four histological variances: a cellular ependymoma, a clear cell ependymoma, a papillary ependymoma, and an tanycytic ependymoma [34]. Cellular ependymoma is a term that has disappeared from the new WHO 2016 standard and is classified as either classical or anaplastic or as belonging to the *RELA* fusion-positive subtype. A cellular ependymoma is a common intramedullary subtype, but its incidence is rare. A cellular ependymoma is identified by hypercellularity composed of pseudorosettes, not true ependymal rosettes [24,35]. A clear cell ependymoma consists of cells with the oligodendroglial phenotype and perinuclear halo [36]. This variant is associated with a worse prognosis [37]. Papillary brain tumors are rare brain tumors that are found more often in the brain than in the spinal cord. A papillary ependymoma is characterized by a papillary structure consisting of single or multiple layers of cubic cells. Additionally, it is observed to have immunohistochemical features including pseudorosettes and no basement membrane [38]. A tanycytic ependymoma is a rare subtype derived from tanycyte, which is usually located more in the spinal cord than in the brain. The development of this tumor is a streaming process of elongated cells that lack the typical ependymal rosettes and obscure perivascular rosettes [39]. A *RELA* fusion-positive ependymoma is recognized as WHO II or III through the 2016 WHO classification update of CNS tumors. *RELA* fusion-positive subtypes account for approximately 70% of the supratentorial ependymomas found in both children and adults [40]. The *RELA* fusion-positive subtype is most commonly observed with *C11orf95-RELA* fusion [41], resulting in constitutive activation of the NF-κB pathway [42]. A WHO grade III anaplastic ependymoma is a variant tumor that accounts for approximately 8.6 to 11.5% of all ependymoma types [43]. An anaplastic ependymoma is mainly found in the posterior fossa of infants and children [44]. It is a malignant tumor that grows faster in the base of the brain and rarely in the spinal cord compared to other types [24]. It has been reported that these tumors correlate with poor outcomes in posterior fossa tumors due to their higher proliferation and aggressiveness [45].

Ependymoma statistics showed that the prevalence of ependymomas was highest for children, and the survival rates were comparatively lower for children [33,46]. Ependymomas accounted for 5.7% of primary brain or CNS tumors for children, among 16,366 incidences. The percentage of brain or CNS tumors that were ependymomas decreased with increasing age, with a prevalence of 4% for 15–19 years and 1.9% for adults (>19 years) (Table 2). From a statistical report, it was found that ependymoma patients aged 20–44 years showed high survival rates (91%) [33,46], whereas the survival rates were lowest for those aged 75+ years (57.8%) and for children aged 0–19 years (75.2%, Table 3).

## 4. A Molecular Classification of Ependymomas Using DNA Methylation Profiling

Traditionally, CNS tumors have been classified according to histopathological characteristics [24]. However, a molecular classification system has been suggested as a novel classification method based on DNA methylation and gene expression profiling studies using advancing genome sequencing technology [41]. Epigenetic changes, such as DNA methylation and chromothripsis, are recognized as universal hallmarks of tumorigenesis, and the DNA methylation pattern of tumors is specific and highly stable, making it particularly suitable for tumor classification [47]. Ependymomas consists of the following three anatomical compartments: the supratentorial (ST), the posterior fossa (PF), and the spinal cord [48,49,50]. Therefore, molecular classification based on the association with the anatomical location, histological, and genetic changes allows the nine subgroups of ependymomas to be distinguished (Table 4) [9,41,51].

Molecular classification using anatomical compartments and the DNA methylation profile newly classifies ependymomas including the myxopapillary ependymoma (MPE), the subependymoma (SE), the (anaplastic) ependymoma (EPN), and the *RELA* fusion-positive ependymoma (EPN-RELA) [41]. All the histologically classified subependymomas and myxopapillary ependymomas undeniably belong to the SE and MPE groups classified by the following molecular classification: SP-SE, PF-SE, ST-SE, and SP-MPE. As a result of DNA methylation profiling, SEs usually showed a loss of chromosome 19, which was found most frequently in PF-SE (79%) but also in ST-SE (50%) and SP-SE (40%) [52]. In addition, the partial loss of chromosome 6 was frequently observed in SP-SE and PF-SE. In particular, *TERT*-promoter mutations/loss of chromosome 6 were frequently found in PF-SE tumors, and these phenotypes have been suggested as useful markers for the use of intended therapy with respect to an increased risk of recurrence [53]. The WHO group II ependymoma or III anaplastic ependymoma has multiple cases that are often difficult to distinguish [54]. These groups are divided into SP-EPN, PF-EPN-A, and PF-EPN-B according to molecular classification. Posterior fossa group A (PF-EPN-A) tumors are mainly diagnosed in infants and children and have a higher recurrence rate than posterior fossa group B (PF-EPN-B). PF-EPN-B tumors are mainly found in young adults and have a better prognosis than PF-EPN-A [49,50]. The molecular groups remaining in the supratentorial compartment are the ependymomas with an *RELA* fusion and *YAP1* fusion genes [41,51]. The *RELA* fusion ependymoma, named ST-EPN-RELA, is the result of a fusion between the chromosome 11 open reading frame (C11ORF95) and the v-rel avian reticuloendotheliosis viral oncogene homolog A (*RELA*) [42]. This fusion complex has been shown to serve as an oncogenic transcription factor by acting on chromatin and to induce ependymoma development in mouse models [42,55]. Moreover, this fusion complex has been reported to induce tumorigenesis when aberrantly expressed in neural stem cells [42]. The remaining supratentorial variant is called ST-EPN-YAP1 and is mainly found in children. ST-EPN-YAP1 fusions have been reported with both *YAP1-MAMLD1* and *YAP1-FAM118B*, but their functions are still unclear. It is not known whether each fusion complex could have a similar function, but MAMLD1, a member of the mastermind gene family, is known to be an important regulator of notch signaling transcription and the p53 tumor suppressor pathway [56]. Furthermore, it has been reported that FAM118B acts as a tumor suppressor and reduces the proliferation of HELA cells [57].

Accurate histopathological diagnosis of ependymomas according to the WHO classification is often difficult because it is not clear whether the graded components of the tumor affect the overall biological behavior. These subgroups are categorized for unique genetic and epigenetic features and may be classified and diagnosed much more accurately than those assessed by the WHO grades alone.

## 5. Metabolism of Brain Tumor

Malignant brain tumors are a severe problem for both children and adults [58]. The brain usually obtains almost all its energy from glucose metabolism [58,59,60]. Most of the glucose is metabolized to pyruvic acid and enters into mitochondria to generate energy through the TCA cycle [60]. The normal brain is not limited to glucose metabolism. When blood sugar levels decrease, ketone bodies are oxidized and used for energy metabolism [61,62]. Ketone body metabolism is a physiologically adaptive response to preventing excessive protein use and maintaining proper brain function [62]. Unlike glucose, ketone bodies go directly into the mitochondria to be metabolized without going through glycolysis. However, malignant brain tumors rely solely on glucose for energy [63]. Enhanced glycolysis produces excess lactic acid. The produced lactic acid is returned to glucose through the Cori cycle, where it again participates in tumor metabolism [64]. Except for glycolysis dependence, brain tumors have abnormalities in the number and function of mitochondria. Therefore, it is difficult to use ketone bodies for energy metabolism [65]. Given the energy metabolism of these brain tumors, a glucose–ketone-restricted diet naturally inhibits glycolysis and tumor growth by lowering the circulating glucose levels, while enhancing the activity of normal tissues through ketone body metabolism [63,66]. The ketogenic diet (KD) is an effective treatment for fatal infantile brain tumors that have been used for decades as a high-fat, low-carb diet. In a KD-fed mouse brain tumor model, it was confirmed that the inhibition of brain tumor growth was directly related to decreased blood glucose levels and increased blood ketone body levels [63,67,68]. Angiogenesis is known to be closely related to the metabolic activity of human gliomas. It was confirmed that it has an antiangiogenic effect by reducing the tumor energy metabolism for a KD [67,68]. Moreover, the restriction of tumor metabolism induces pro-apoptotic effects as well as anti-inflammatory effects [69]. This treatment may be very effective with a limited diet after the tumor cells have been physiologically weakened using conventional treatment regimens such as surgery, radiation, and drug therapy [70]. The goal of this novel brain tumor treatment is to establish a new strategy for human brain tumors by improving the activity of normal brain cells while metabolically inhibiting tumor cells.

## 6. Mechanism and Metastasis of Ependymoma

**Figure 1:**
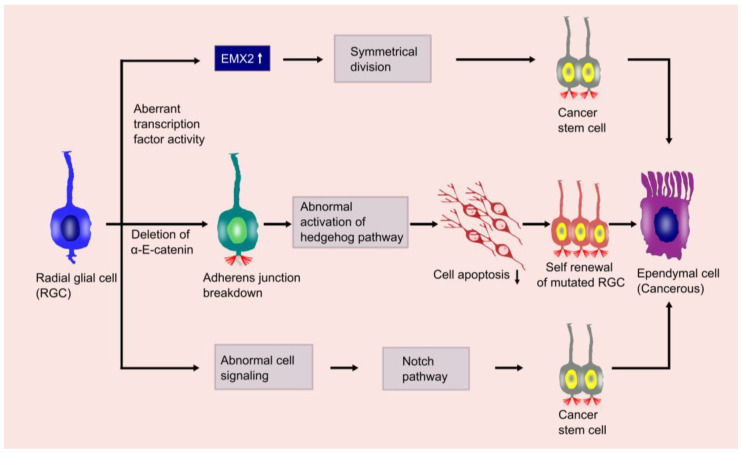
Tumorigenesis of ependymal cells. This figure illustrates the possible mechanism of tumorigenesis of ependymal cells from the radial glial cell (RGC). RGC mutation by aberrant transcription factor activity, adherens junction breakdown, or abnormal cell signaling transforms it into a cancer stem cell (CSC).

Tumorigenesis is a complex multistep process that transforms a normal cell into malignant cells or cancer stem cells (CSCs). The process comprises the following three stages: initiation, promotion, and progression. This multistep process involves activation of the oncogene, inactivation of a tumor suppressor gene, as well as epigenetic phenomena that alter gene expression. The initial cell mutation, due to either spontaneous or carcinogenic exposure, causes the initiation of a tumor. During tumor promotion, the transformed cells undergo selective clonal expansion and give rise to a large number of mutated daughter cells. In tumor progression, the benign tumor becomes more aggressive and expresses a malignant phenotype. Traditionally, ependymomas are thought to originate from ependymal cells that line the ventricles and the central canal of the spinal cord. A study conducted by Taylor et al. demonstrated that RGCs were ependymoma stem cells or the root cells of an ependymoma [18]. They conducted the study by reproducing the disease in a murine model by xeno-transplantation of human ependymal cells and observed the similar CD133+/Nestin+/RC2+/BLBP+ immunophenotype to RGCs. RGCs are multipotent and self-renewing progenitor cells in the brain. They have the ability to proliferate into neurons and glial cells, including astrocytes, oligodendrocytes, and ependymal cells [71]. A recent study demonstrated that the oncogene EPH receptor B2 (EPHB2) could induce an ependymoma by converting an RGC in the forebrain into a CSC [11,48]. They also found that the conversion of RGCs by EPHB2 does not occur in the hindbrain or the spinal cord, suggesting that the genetic events of ependymomas are site-specific. However, aberrant transcription factor activity such as empty spiracles homeobox 2 (*EMX2*), breakage of the adherence gene αE-Catenin at the apical cell junction, and deregulation of notch signaling pathways have been found to convert the RGC behavior and provoke ependymoma activity [72,73]. RGCs and neuroepithelial cells (NECs) can divide symmetrically or asymmetrically. Symmetrical division generates identical cells where both of the daughter cells can proliferate. In contrast, asymmetric division produces a stem cell and a non-stem cell. The entire process is firmly controlled and any anomalies in this process result in the progression of a CSC. *EMX2* and *PAX6* genes strictly maintain the balance between the two division types to regulate stem cell proliferation. Expression of EMX2 can cause symmetrical division, whereas expression of EMX2 with PAX6 can cause asymmetrical division. An abnormal and sustained expression of EMX2 promotes symmetrical division, resulting in an increased RG cell number and increased stem cell proliferation. Uncontrolled stem cells emerge as CSCs and cause neoplasm [72,74]. Transcription factor EMX2′s upregulation is notably observed in supratentorial ependymomas. Interestingly, due to the context-dependent nature of stem cells, the expression of EMX2 within the periventricular region of the adult telencephalon acts as a negative regulator of symmetrical cell division in adult neural stem cells [72,75]. Adherens junctions are found below the apical plasma membrane of RGCs and studies have revealed that the disruption of these junctions also contributes to tumorigenesis [76]. Loss of the α-E-Catenin gene from progenitor cells leads to the disruption of these junctions [77] and the abnormal activation of the Hedgehog (Hh) signaling pathway [78]. In tumorigenesis, Hh signaling pathway activation enhances cell proliferation and reduces cell apoptosis (Figure 1) [79].

Tumorigenesis also involves the deregulation of different pathways, such as the Notch and EPHB-Ephrin signaling pathways. The deregulation of these pathways indicates supratentorial ependymomas [48,80]. In neural development, Notch signaling plays an essential role, including proteolytic cleavage that functions in transcription regulation [81]. The deregulation of Notch signaling is assumed to be involved in ependymal cell tumorigenesis, particularly in supratentorial ependymomas [80,82]. A recent study conducted by Taciani et al. reports that, although notch signaling is especially activated in the ST-EPN-RELA subgroup, the activation of notch signaling is not required for cell proliferation and survival for ST-EPN-RELA, rather it is associated with maintaining the expression of CSC marker genes in ST-EPN-RELA [83]. Methods of notch signaling deregulation include the upregulation of ligands such as Jagged canonical notch ligand 1/2 (JAG 1/2), Notch receptor 1/2, target genes MYC proto-oncogene (*c-MYC*), hes family bHLH transcription factor 1/5 (*HES1/5*), and hes related family bHLH transcription factor with YRPW motif 2 (*HEY2*) as well as the downregulation of repressor tumor suppressor F-box and the WD repeat domain containing 7 (*FBXW7*) gene.

**Figure 2:**
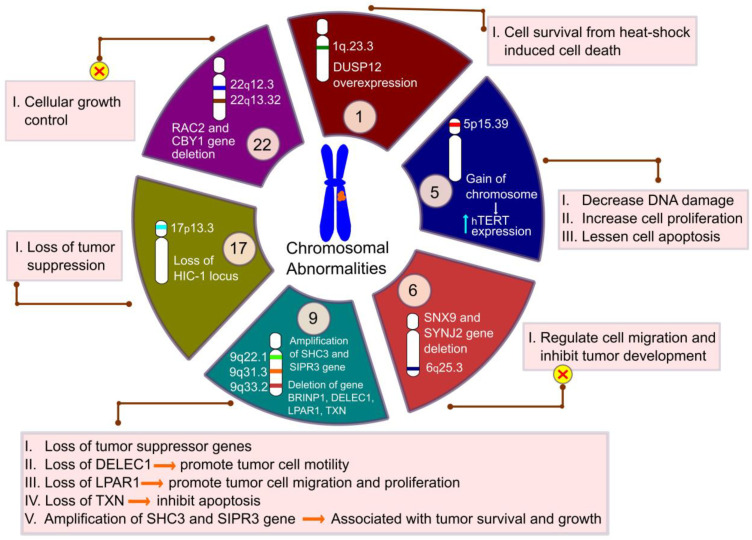
Chromosomal abnormalities in ependymoma. This figure represents the loss or gain of the chromosomal region. Deletion or overexpression of some important genes within those regions plays an important role in tumor formation.

Chromosomal abnormalities play a remarkable role in ependymomas (Figure 2). Translocation, partial loss, or monosomy 22 is frequently observed in 26–71% of cases of ependymomas [80]. The deletion of the RAC family small GTPase 2 (*RAC2*) and chibby family member 1 (*CBY1*) gene in the chromosome 22q12.3–22q13.33 region are observed in 38% of intracranial ependymomas [84]. Transcriptional inactivation of the *CBY1* gene is also observed in 60% of intracranial ependymomas. Deletion of the *RAC2* gene affects cellular growth control and induces tumor formation. The gain of chromosomes 1q, a momentous tumor aggressiveness prognosticator, is often detected in pediatric intracranial ependymomas [85]. In intracranial ependymomas, Mendrzyk et al. have revealed the two most frequent chromosomal gains of 1q to occur in the 1q21.3–23.1 region as well as in the 1q31.1–32.1 region [86]. They have also identified the overexpression of oncogene dual specificity phosphatase 12 (*DUSP12*) in the 1q23.3 region, which is concerned with tumor cell survival [86,87]. The removal of tumor suppressor genes sorting nexin 9 (*SNX9*) and synaptojanin 2 (*SYNJ2*) in the 6q25.3 location of chromosome 6 disturbs their functions, including the regulation of cell migration from the primary tumor site to distant organs and the inhibition of tumor development [88]. Furthermore, the gain of chromosome 5 at the 5p15.33 location elevates the human telomerase reverse transcriptase (*hTERT*) gene expression, which is involved with telomerase activity [89]. The elevated expression of *hTERT* promotes ependymoma development by decreasing DNA damage, increasing cell proliferation, and lessening cell apoptosis [89,90]. Tumor suppressor gene hyper-methylated in cancer 1 (*HIC-1*) is hyper-methylated and downregulated in 81–83% of ependymomas. The loss of the *HIC-1* locus at the 17p13.3 chromosomal region is frequently observed in ependymoma patients [91]. Chromosome 9 abnormalities are also observed in adults along with pediatric ependymomas, as reported in a microsatellite analysis by Schneider et al. [92]. Deletion of the chromosome at the 9q31–33.2 region includes the loss of the tumor suppressor gene bone morphogenetic protein/retinoic acid inducible neural-specific protein 1 (*BRINP1*), deleted in esophageal cancer 1 (*DELEC1*), lysophosphatidic acid receptor 1 (*LPAR1*), and thioredoxin (*TXN*). Magrassi et al. also reported the chromosomal 9 gain at the 9q22.1 region that was identified as the co-amplification of SHC-transforming protein 3 (*SHC3*) and sphingosine-1-phosphate receptor 3 (*S1PR3*, also called *EDG3*) in 60% of ependymomas [93]. The activity of the *SHC3* gene is entailed with the signaling pathway of transmembrane receptor protein tyrosine kinase, and functional dysregulation in the expression of this gene induces factors associated with the survival of anaplastic astrocytomas and glioblastomas [94]. While the *S1PR3* gene expresses in endothelial cells and contributes to angiogenesis regulation, their study suggested that co-amplification of the *SHC3* and *S1PR3* genes is connected with ependymomal tumor survival and growth in both posterior fossa and spinal cord ependymomas [93,95]. *HOXB13*, a member of the homeobox (HOX) gene family, at the 17q21.32 region of chromosome 17, is overexpressed in myxopapillary ependymomas. HOXB13 overexpression is a unique feature of myxopapillary ependymomas in pediatric patients, identified by Barton et al. [96,97]. An elevated expression of HOXB13 enhances tumor cell proliferation and also its migration to distant organs [98]. Their study also reported the upregulation of some genes, including neurofilament light polypeptide 68 kDa (NEFL) and platelet-derived growth factor receptor A (PDGFRA) in myxopapillary ependymomas through immunohistochemistry (IHC) analysis. PDGFRA overexpression is also observed in intracranial ependymomas. PDGFRA plays an important role in angiogenesis during ependymoma development [99].

Moreover, 112 aberrantly expressed genes that influence ependymoma development were identified by Suarez-Merino et al. Their study includes the overexpression of oncogene Wnt family member 5A (WNT5A), tumor protein p63 (TP63), msh homeobox 1 (MSX1), insulin-like growth factor binding protein 2 (IGFBP2), chromobox 7 (CBX7), collagen type IV alpha 1 chain (COL4A1), WEE1 G2 checkpoint kinase (WEE1), leucine rich repeat neuronal 2 (LRRN2), angiogenesis factor vascular endothelial growth factor A (VEGFA), fibronectin 1 (FN1), and transcription factor ZIC1 [100]. Among these genes, overexpression of Wnt5A, a Wnt family member, enhances tumor cell motility, invasion, inflammation, and metastasis to other organs [101]. Overexpression of IGFBP2, an insulin-like growth factor (IGF) system regulator, exhibits a potential role in glioma progression and metastasis [102]. COL4A1 and LRRN2 also have significant roles in glioma progression [103,104]. The upregulation of VEGFA, a VEGF family member, has a remarkable role in tumor angiogenesis [105]. The *FN1* gene encodes the fibronectin glycoprotein that promotes tumor migration and invasion and suppresses cell apoptosis via the focal adhesion kinase (FAK) pathway [106]. ZIC1 is a member of the ZIC gene family that is highly expressed in the cerebellum [107]. The downregulation of genes including *EB1* and Schwannomin-interacting protein 1 (*SCHIP-1*) was also observed, along with the *NPC1*, *TJ2*, *RAB40B*, and *SH3GL3* genes [100].

**Figure 3:**
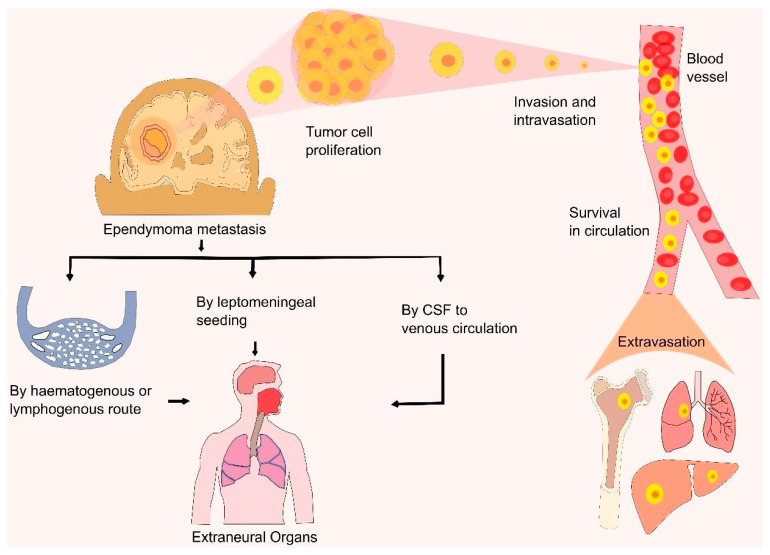
Metastasis of ependymomas. This figure illustrates the dissemination of an ependymal tumor cell from its primary site to the secondary site. The ependymoma metastasizes to an extraneural organ (e.g., skull, lungs, liver, bone, or lymph nodes) via several routes, including the haematogenous or lymphogenous routes. When tumor cells separate from their primary tumor, they intravasate into the blood vessels, circulate, and extravasate into their target organs.

The primary ependymoma tumor in the CNS mostly arises from the spinal cord central canal, filum terminal, cauda equine, or ventricular wall [108]. However, dissemination of ependymomas from the primary site has been reported in several cases and most of them were first elaborately described in studies from the early 1950s [109,110,111]. Researchers found that, in pediatric patients, 9–20% of ependymomas metastasize from their primary site and the survival rate of patients with metastatic ependymomas varies widely, ranging from 0 to 80% [112,113]. When tumor cells lose their adhesiveness, they escape from their primary site and metastasis occurs. In most cases, ependymomas metastasize through the haematogenous route or the lymphogenous route [114]. However, tumor tissue dissemination via the CSF was also reported in anaplastic ependymomas and most benign ependymomas [114,115]. During tumor resection surgery, rupture of the blood–brain barrier (BBB) results in CSF dissemination, allowing tumor cells to enter the venous system and spread to extracranial sites. Danial et al. reported a case of pulmonary metastasis via CSF seeding, which causes tumor cells to spread through the haematogenous route, resulting in tumor cell seeding to the lungs [116]. Leptomeningeal seeding from CSF was also observed in many cases before the progression of extraneural metastasis [114]. Due to the breaking of the defensive anatomy of the CNS during ependymoma resection surgery, the dissemination of tumor cells in extraneural organs also occurs. Tumor cells invade, intravasate into the blood vessels, circulate, and extravasate into their target organs. Children with intracranial ependymomas present rare primary metastatic disease [114]. Despite the lack of detrimental features in the histology, myxopapillary ependymomas may develop in CSF propagation [117]. Research conducted by Zhu et al. reported several cases of MPE dissemination, including the sacral canal, spinal cord, cerebellum, and fourth ventricle via the CSF route [115,118]. Chakraborti et al. reviewed 19 cases and reported the intracranial dissemination of MPE at different locations such as the brainstem, cerebellum, and telencephalon [115,119]. Another recent review by Gray et al. showed 15 cases of disseminated anaplastic ependymomas to the extraneural sites including the paratoid glands, lymph nodes, lungs, scalp, and liver, where most primary tumors arise from the supratentorial region [114]. The determination of the mechanism of ependymomas and the observation of their metastasis process by several researchers helps to find new therapeutic and treatment procedures for ependymomas (Figure 3).

## 7. Signs and Symptoms

The clinical stage of an ependymoma reflects the size and site of the tumor [15]. The clinical prominence of an myxopapillary ependymoma is undefined and sometimes physicians fail to identify it as an ependymoma [41]. The main indication of these tumors is back and leg pain and physicians often diagnose it as radicular pain. However, the signs and symptoms of myxopapillary ependymomas also include numbness and sensory, motor, gait, and urinary dysfunction [120,121]. The clinical features of ependymal tumors are non-specific, depending on the location, size, and severity of the tumor [11,15]. Anaplastic ependymomas generate signs and symptoms more swiftly [15]. Intracranial ependymomas also cause nonspecific presentations such as headaches, irritability, nausea, vomiting, and lethargy [122]. Besides these signs and symptoms, intracranial ependymomas show some additional signs such as late growth and an enlargement of the head, called macrocrania, in children [123]. Supratentorial ependymomas may be accompanied by headache, seizures, and focal neural symptoms [124]. The gradual contraction of the posterior fossa structure results in hemiparesis, visual disturbances, ataxia, neck pain, and dizziness [125].

## 8. Diagnosis

The methods for diagnosing an ependymoma mainly include MRI and CT scans. The primary imaging procedure used for diagnosing an ependymoma is MRI, as it has higher soft-tissue contrast [126,127,128]. For children, in the case of emergency, a CT scan is often performed before an MRI scan [123,129] because a CT scan can provide better calcification information than an MRI scan. However, the characteristics that make MRI the primary imaging method for ependymomas are the multiplanar imaging systems, the lack of ionizing radiation, and its superior tissue contrast ability [20,127]. An ependymoma presents a well-defined lesion with varying degrees of reverse enlargement, more remarkable in anaplastic tumors, and may be absent in subependymomas on MRI or CT scanning [15]. Subependymomas are almost only found in adults and have low proliferation indicators, with better prognosis after complete surgical detection [130]. The solid tissues of ependymomas are generally iso- to hypo-dense with cystic areas in a CT scan, iso- to hypo-intense in a T1-weighted MRI, iso- to hyperintense in a T2-weighted MRI, and iso- to hyper-intense in conventional MRI sequences, called fluid-attenuated inversion recovery (FLAIR) sequences [21,129]. Advanced MRI techniques, including diffusion-weighted imaging, proton magnetic resonance (MR) spectroscopy, susceptibility-weighted perfusion, and dynamic contrast-enhanced MR imaging, help physicians to differentiate between the different types of brain tumor more efficiently [131,132]. Diffusion-weighted imaging is helpful for understanding the differences between diverse sorts of posterior fossa tumors in children including ependymomas, medulloblastomas, and pilocytic astrocytomas [133]. Susceptibility-weighted imaging and MR spectroscopy are used to recognize post-treatment changes and help if there is any doubt in the neoplastic characteristics in the initial lesion [129]. Proton MR spectroscopy exhibits higher choline and diminished N-acetylaspartate levels [11,126]. Perfusion MRI may exhibit some prognostic value and a higher rate of cerebral blood volume [129,134]. Diffusion tensor imaging and diffusion tensor tractography along with MRI can be used to identify intramedullary spinal ependymoma for better surgical management [135]. As intracranial ependymomas can move into CSF spaces, patients with these tumors must be assessed periodically with an MRI scan of the whole neuraxis [136]. Genome and epigenome comprehensive profile of ependymal tumors should identify more molecular deficiencies in each of these identified symptoms as well as its clinical concern [137]. Current studies have predicted that surgery, radiation therapy, and chemotherapy are the crucial treatment for patients with an ependymoma [138]. Recent and future scientific endeavors require a comprehensive evaluation of all these evolving molecular phenomena and the development of reasonable therapeutic goals [137].

## 9. Therapeutic Advances

**Figure 4:**
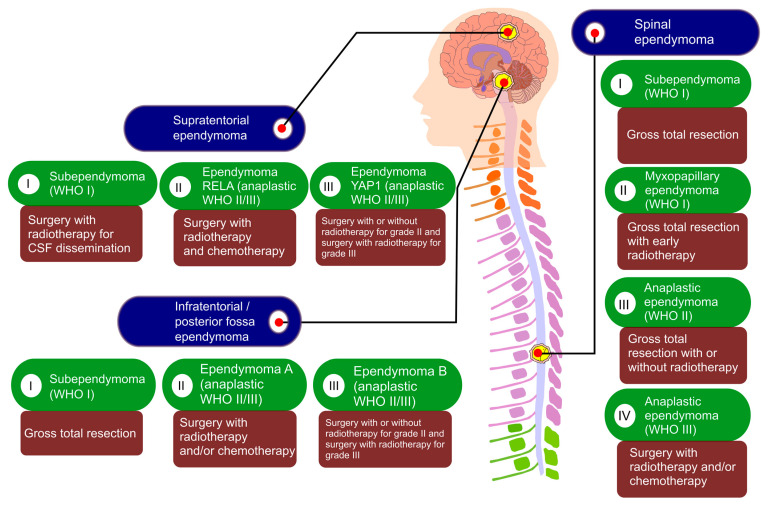
Possible therapeutic approaches. This figure represents the different types of ependymomas depending on their positions and WHO’s classifications, and their therapeutic advances. Surgical resection is considered a primary therapeutic approach, while, in many cases, RT with surgery shows a greater response. Chemotherapy can be also used for anaplastic ependymomas as a therapeutic method. For subependymomas, only surgery can be curative, RT is essential for recurrent subependymomas. Although in some cases, it is important to deliver RT on the tumor region after surgery for myxopapillary ependymomas, most of the cases show great results only with surgery. In WHO II ependymomas, targeted treatment is to acquire GTR, RT is required after STR. For anaplastic ependymomas, the suggested therapy is surgery with RT. However, in some exceptional cases such as recurrent anaplastic ependymoma or very young children, chemotherapy is required after surgery with or without RT because RT has toxic effects on children’s developing brains and sometimes surgery with RT fails to show effective results in recurrent disease. However, a lack of an established targeted chemotherapy makes surgery and RT primary treatment options [16,139].

Staging systems are helpful for cancer management. However, no standard staging systems exist for managing ependymomas. The possible therapeutics of ependymomas mainly rely on the patient’s age, molecular subgroup, histological grade, and whether the ependymoma has spread [139]. Basically, the possible therapeutic approaches of ependymomas include chemotherapy, surgery, and RT (Figure 4), where surgery and RT are considered to be the primary therapeutic advances.

### 9.1. Surgery

The most important primary treatment of an ependymoma is surgery [140]. Surgical treatment sets up a diagnosis, switching hydrocephalus, and restoring the CSF flow [141]. With a gross total resection (GTR), 60–89% of patients outlive 5 years and 70% of patients outlive 10 years. On the contrary, with an incomplete resection, 21–46% of patients outlive 5 years and 11% of patients outlive 10 years [140,142]. In a case study, 56% of a local control (LC) rate of 10 years after a GTR and 92% of a LC rate of 10 years after a GTR with RT are reported. The 10-year LC rate for the subtotal resection (STR) is 0%, whereas it is 65% when patients receive RT after an STR [143]. Other studies have revealed that, in patients with a spinal myxopapillary ependymoma, after a GTR, the average 5-year overall survival (OS) rate is 94.99 ± 3.87%, and the 10-year OS rate is 92.31 ± 5.73% [144]. The complex position of the ependymoma is the main cause of incomplete resection [142]. The tumors that bind vascular systems, the ventricular surface or cranial nerves, and intrinsic brainstem tumors are reluctant to be fully resected [141]. Follow-up surgery is required to obtain complete resection as it increases the chances of survival [144,145]. The complications related to incomplete resection include CSF dissemination and recurrent ependymomas [121]. Due to the risk of CSF dissemination for patients with a grade II and III ependymoma, disease staging requires MRI of the whole neuraxis and CSF analysis. If not acquired at the very beginning of surgery, CSF analysis through lumbar puncture must be postponed for at least two weeks following surgery to prevent confounding results linked to postoperative changes [146]. For certain patients with a myxopapillary ependymoma or a subset of patients with a supratentorial ependymoma, surgical resection alone can be remedial [147]. Postoperative radiotherapy is essential for WHO I and II ependymomas when a GTR cannot be obtained and is also important for WHO III anaplastic ependymomas [121]. Stereotactic neurosurgical approaches are driven by CT scans and MRI as well as advances in neuro-anesthesia, and postoperative intensive care helps the physicians to provide better surgical treatment [147].

### 9.2. Radiotherapy

RT is also one of the standard treatments for ependymomas, and it has historically demonstrated great promise for low-grade lesions, mainly after a gross total resection [129,148]. A variety of retrospective, non-randomized studies reported an excellent survival rate with surgery and RT compared to just surgery [139,149]. Although a gross total resection with RT or a gross total resection alone is a possible treatment for adult patients with WHO grade I or II ependymomas, RT is required for patients with WHO grade III ependymomas, even after a gross total resection [139]. In non-disseminated children who are more than one year old, RT is usually suggested for some of WHO grade II and all of WHO grade III ependymoma resections [11,41]. For grade I myxopapillary ependymomas, surgery is usually curative. However, for certain patients with myxopapillary ependymomas, it is required to provide RT on tumor regions. For grade II ependymomas, the proficiency of RT is still controversial after a GTR [147,150]. There is no confirmed evidence about the perfect dose of RT; nevertheless, the suggested effective dose is at least 45 Gy, where the current standard radiation dose is 50–60 Gy [142,151]. The dose recommendation for low-grade tumors is 54 Gy in 30 fractions and for high-grade tumors is 59.4 Gy in 33 fractions [152,153,154]. In patients with anaplastic ependymomas, regional or focal RT with a dose of up to 60 Gy is effective and comparatively harmless when the disseminated disease is not present [155]. Although craniospinal irradiation with a dose of 36 Gy, which boosts up to 59.4 Gy, is suggested for patients with disseminated disease, the proficiency of craniospinal irradiation on the survival rate is unproven [154,156,157]. However, nearly all researchers suggested focal RT instead of craniospinal irradiation when disseminated disease is absent [11]. As conventional photon RT has a long-term side effect on children’s developing brain, proton beam therapy (PBT) may be useful for young children with tumors where the complete resection of these tumors is not always possible, and a higher dose of RT is needed near critical structures in the CNS [158,159]. The unique characteristics of PBT are to diminish the ionizing radiation dose stored in uninvolved CNS tissue, mainly by dropping the dose suddenly and significantly reducing the exit dose. It prevents the toxic effect of ionizing radiation related to hearing, endocrine, and cognitive functions [123]. Other modern RT includes intensity-modulated radiotherapy (IMRT) and volumetric modulated arc therapy (VMAT) [160]. IMRT can deliver radiation on cancer cells more accurately and spare the healthy cells around it. A study has reported a 5-year OS rate of 40.6% for patients who are less than 3 years old with anaplastic ependymomas after receiving IMRT followed by surgery. This study concluded that IMRT exhibits great local control and less toxicity than 3D conformal RT [161]. In the perception of fixed-field or “step and shoot” IMRT, radiation is dispatched with a static beam position, whereas VMAT focuses on intensity-modulated IMRT delivery in an uninterrupted manner during the treatment and the treatment time gambles as a consequence [160]. However, the usefulness of modern PBT, IMRT, and VMAT in the treatment of ependymomas is still a field of active research.

### 9.3. Chemotherapy

Though chemotherapy may be a useful treatment for patients with tumors where radiation or surgery is not feasible, the efficacy of chemotherapy on ependymomas is controversial [41,129]. As RT might exert neurotoxic effects, young children can go through chemotherapy for treatment to avoid or delay RT [123]. Chemotherapy is suggested after the first surgery attempt for young children with residual disease [8]. The immediate effectiveness of chemotherapy for adult patients diagnosed with ependymomas has been researched retrospectively [11]. In adult patients with recurrent disease, it has been retrospectively observed that platinum-based chemotherapy regimens tend to introduce higher tumor response rates with lower progression rates than nitrosourea-based regimens [162]. Cisplatin therapy may be more helpful than carboplatin in recurrent childhood ependymomas, which has not been exhibited in adult patients [141,147].

There is observational evidence of chemotherapy regimens against brain tumors along with idarubicin, irinotecan, and combination therapy with tamoxifen and isotretinoin [163]. Idarubicin (IDA) belongs to the class of anthracycline antibiotics and has the anticancer effect that inhibits cell division by preventing DNA and RNA synthesis [164]. Unlike other anthracycline anticancer drugs, IDA is highly lipophilic due to the lack of a methoxy group at the fourth position [165], which increases its chances of penetrating brain tissue [166]. However, because IDA does not efficiently cross the blood–brain barrier, the therapeutic effect was not sufficient for medulloblastomas, brain tumors, or brainstem tumors in a phase II trial in patients with recurrent brain tumors [167]. Irinotecan is structurally similar to camptothecin and exhibits anticancer activity by binding to the topoisomerase I-DNA complex and inducing DNA chain damage [168]. In general, irinotecan is being tested in clinical trials for brain tumors as a combination therapy rather than as a monotherapy. It has been demonstrated that irinotecan has antiangiogenic effects by indirectly inhibiting the accumulation of HIF1α [169]. The SFCE-RAPIRI Phase one study has advanced a therapeutic strategy targeting the mTOR/HIF1α pathway using a combination therapy of rapamycin (an mTOR inhibitor) and irinotecan (HIF1α inhibitor) [170]. This drug combination has already shown therapeutic effects in adult patients [171]. In pediatric tumors such as brain tumors, sarcomas, and neuroblastomas, 14 out of 31 patients maintained non-progressive disease for eight weeks using this treatment strategy [170]. In addition, based on a study showing that VEGF is overexpressed in brain tumors, including malignant gliomas, the humanized monoclonal anti-VEGF antibodies bevacizumab and irinotecan were administered to adults with recurrent glioblastomas in phase II studies [172]. Combination therapy showed improvements in response rates and progression-free survival. Based on these encouraging results, the Pediatric Brain Tumor Consortium initiated a phase II study in children with recurrent malignant gliomas. The combination of bevacizumab and irinotecan had only minimal effects in children with recurrent malignant gliomas and brainstem gliomas [173]. Etoposide prevents DNA repair by interacting with the DNA repair enzyme topoisomerase II, causing cytotoxicity [174]. The effects of etoposide have been observed as monotherapy in children with recurrent brain tumors [175]. Oral administration of etoposide had some side effects, but it was well tolerated and cytotoxic, resulting in an obvious therapeutic response. Furthermore, in a phase two study comparing the oral administration of the EGFR inhibitor erlotinib with that of etoposide, a significant effect was observed only with the oral administration of etoposide [176]. Ifosfamide is a parenterally administered alkylating agent similar to cyclophosphamide. A phase II trial was conducted in pediatric patients with various recurrent primary brain tumors. Ifosfamide has been observed to have moderate efficacy in the treatment of recurrent medulloblastomas and ependymomas. However, it is not recommended to establish therapy with ifosfamide in highly advanced brain tumors due to the severe neurotoxicity observed in clinical trials [147,177]. However, adequate information from prospective clinical trials is needed to formulate the proper dose of chemotherapy [178].

The Center for Cancer Genomics protocol 9942 study reported a 5-year event-free survival (EFS) rate of 57% for young patients (3–21 years) after chemotherapy [179]. The Head Start III trial has shown that the 3-year EFS rate is 86 and 27% for supratentorial and infratentorial ependymomas, respectively, for young children (<10 years) after chemotherapy [180]. The Associazione Italiana Ematologia Oncologia Pediatrica estimated a 5-year progression-free survival (PFS) rate of 65.4% after using chemotherapy in children [181]. The ACNS0831 phase III trial has shown that, for young patients (1–21 years), the 3-year EFS rate is 78% after maintenance chemotherapy (vincristine, cisplatin, cyclophosphamide, etoposide) along with RT, and 80% after using any chemotherapy along with RT [182].

Another chemotherapy drug, temozolomide (TMZ), is an alkylating agent that changes DNA by adding a methyl group to adenine at the N3 site and to guanine at the O6 and N7 sites and may be effective for initial CNS tumors [183,184]. As some recurrent anaplastic ependymomas express elevated levels of O6-methylguanine-DNA-methyltransferase, TMZ works against these ependymomas by depleting O-6-methylguanine-DNA methyltransferase [185]. Although some case studies demonstrated the better effectiveness of TMZ in recurrent ependymomas, very few responses were seen in a more extensive retrospective analysis [184]. Another study provides evidence on the greater efficacy of TMZ combined with lapatinib for repeated illness [186].

## 10. Ongoing Trials

The advanced molecular characterization of ependymomas has shown different possible targeted therapies. The European Organization for Nuclear Research directed a phase II trial on the effectiveness of bevacizumab targeting the vascular endothelial growth factor with carboplatin for patients who suffer from the disease repeatedly (NCT01295944) [187]. A phase I clinical trial of 5-fluorouracil (5-FU) exhibited some anti-tumor activity and tolerance of 5-FU [188]. Lapatinib that inhibits ERBB1 and ERBB2 has been studied in phase I and phase II trials [189,190]. Other clinical trials include the assessment of neural epidermal growth factor-like 2 (NELL2) and laminin α2 (LAMA2) markers and *RELA* fusion for posterior fossa and supratentorial tumors, respectively (NCT02265770). Applying marizomib in cases of recurrent ependymomas is also undergoing clinical trials (NCT03727841) [191]. Everolimus (NCT02155920) and 5′-azacitidine with carboplatin (NCT0306021) are being investigated as treatments for recurrent ependymomas [137]. The drug TMZ is used during chemotherapy in stage I to stop the proliferation of tumor cells. It works in intracranial ependymomas through salvage treatment. TMZ performs the alkylation of adenine and guanine residues of DNA, damages the repair mechanism of DNA, and leads to cell death [192]. It actually hits on recurrent chemo-naïve adult patients. It has been suggested that, after the failure of surgery and radiotherapy, TMZ is being considered for possible first-line treatment [11]. The combined usage of this drug works more profoundly. A trial in phage Ⅰ shows effectiveness using this drug (ClinicalTrials.gov Identifier: NCT00004892). TMZ is also on trial in the case of phase II. It kills the tumor cells, suppresses the growth of those cells, halts the cellular division, and overall treats malignant gliomas. This study of the drug is not complete yet (ClinicalTrials.gov Identifier: NCT00498927). For better ependymoma management, additional new trials need to be designed in parallel with or after the completion of ongoing trials.

**Figure 5:**
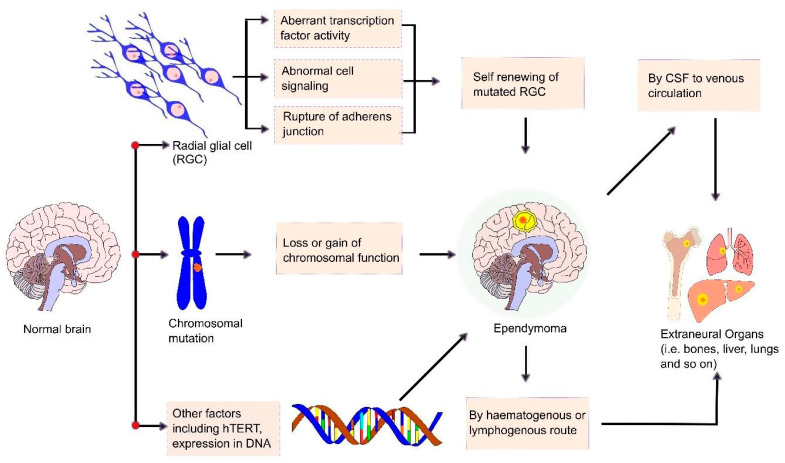
The possible mechanism of ependymomas and their metastasis. Ependymomas arise from the cranial ventricular system or spinal cord. Radial glial cells (RGCs) are thought to be the root cell of ependymomas. RGCs are multi-potent self-renewing progenitor cells in the brain that can proliferate into neurons and glial cells. The mutation of RGCs by aberrant transcription factor activity, adherens junction breakdown or abnormal cell signaling transforms them into a cancer stem cell. Chromosomal abnormalities also play a remarkable role in ependymal tumor formation. When tumor cells separate from their primary tumor, they metastasize to extraneural organs (e.g., skull, lungs, liver, bone, and lymph nodes) via several routes including haematogenous or lymphogenous routes.

Ependymomas are thought to be caused by oncogenetic events that turn normal ependymal cells into tumor phenotypes. The precise nature and order of these genetic events are uncertain. The schematic diagram in Figure 5 depicts the pathophysiology of ependymomas in broad strokes.

## 11. Limitations, Future Prospective, and Conclusions

The management of ependymomas is the main problem, and surgical resection and RT are the ultimate treatment. The approaches can contribute to the effective management of ependymomas. People with grade III ependymomas follow postoperative RT and those who also cannot undergo a complete resection because of the low grade of ependymoma also receive postoperative RT [11]. Chemotherapy has been used as a therapy, though the role of chemotherapy in the management of ependymomas has not been defined properly until now. As a result, the efficacy of chemotherapy is still uncertain. Chemotherapy has been used in younger children instead of RT, but the role of chemotherapy in adult patients has not been studied properly and retrospectively. Though the biological information and molecular classifications of ependymomas have been studied, the management systems for ependymoma remain the most challenging problem. At the same time, because the disease is rare, it is difficult to acquire sufficient data to perform large-scale or randomized clinical trials. Years of research have been carried out on various aspects of the tumor, yet the complete molecular mechanism of the disease is not understood. Therefore, it is still an uphill task to unravel the mystery of the functional interplay. Three distinct types of ependymoma (WHO grade I, II and III) can arise from three different tumor prognostic factors. Each grade of ependymoma has explicit physiological as well as pathological traits. Numerous studies have been conducted to establish a prognosis for the disease. However, the gravity of the disease may depend on factors such as the age and the histology of the affected individual. These multiple factors have made the disease a more difficult problem to solve. Due to poor prognosis, tumor resection using RT could be a solution. Proton therapy is another adjuvant in the case of the management of ependymoma. It is suggested that photon therapy is superior for the management of pediatric ependymomas. This therapy is also favorable for younger patients, and, therefore, this treatment can be followed in the future. This therapy holds bright prospects owing to the reasons mentioned above. Identifying unique molecular features and genomic characteristics may provide new knowledge to develop clinical trials. Along with the treatments, the management of symptoms and their treatment is equally important to rescue patients from this neurological problem [11]. It is important to continue to study ependymoma to further unveil the histopathological features, diagnosis, prognosis, and probable efficacious therapeutic measures of it. It is noted that this paper focuses on the overall features of ependymoma rather than focusing particularly on age groups or histopathological features, treatment, recent advances, and management [10,44,193].

However, some ongoing trials to overcome the difficulties may pave the way to a new horizon in medical treatment. The implementation of systematic postoperative irradiation over the past 20 years significantly extends the life span duration along with disease control and outstanding results [8]. The recently identified, multiple independent genomic profiling makes it easier to identify different subgroups of ependymomas based on clinical and molecular attributions [8]. Remarkable advances have been taken place in understanding the biology and oncogenesis of ependymomas. Surgery and irradiation remain the cornerstone of treatment [23].

Though some recent advances spur up, the management and treatment of ependymoma still remain challenging. The establishment of biomarkers to provide different subgroups has been treated with skepticism. Numerous studies should be conducted to further understand the underlying biology of the targeted therapies to establish a treatment option. Additionally, as this brain disease is predominant in children, age should be emphasized more in further studies.

## Figures and Tables

**Table 1:** Classification and histopathological features of ependymomas that are provided by World Health Organization 2016 [24].

WHO Classifications	Tumor Names	Characteristics
WHO grade I	Myxopapillary ependymoma	- Accounts for approximately 10% - Occurs in young adults - Usually no atypia, no / low mitotic activity - Slow growing ependymal glioma composed of cells arranged in a papillary pattern - Presence of GFAP expression - Lacking cytokeratin expression
	Subependymoma	- Account for about 8% of all ependymomas - Affects middle-aged to elderly adults, occasionally children - Benign, non-invasive, slow growing - No / low mitotic activity - Contain isomorphic nuclei - Most commonly found in the fourth ventricle
WHO grade II	Ependymoma	- Account for about 3 to 9% of ependymomas - Appear at any age - No / rare mitotic activity - Monomorphic nuclei - Infratentorial ependymomas are more common in children - Spinal tumors are more common in adults (30–50 years) - Supratentorial ependymomas affect both children and adults - Four histological subtypes: cellular ependymoma, clear cell ependymoma, papillary ependymoma, and tanycytic ependymoma
WHO grade II or III	*RELA* fusion-positive ependymoma	- Account for 70% of childhood supratentorial ependymomas, with 23% in adults - Usually *C11orf95-RELA* fusion - Fusion leads to constitutive activation of NF kappa B pathway
WHO grade III	Anaplastic ependymoma	- Account for approximately 8.6 to 11.5% - Usually occurs in infants and children - Marked hypercellularity, cellular, and nuclear pleomorphism, and brisk mitotic activity - Higher aggressiveness and invasiveness

**Table 2:** Prevalence of ependymomas among the incidence of primary brain or central nervous system (CNS) tumors [33,46].

Age	Incidence of Primary Brain or CNS Tumors	Percentage of Ependymoma
0–14 years (children)	16,366	5.7%
15–19 years	6747	4%
>19 years (Adults)	356,858	1.9%

**Table 3:** Survival rates of ependymoma [33,46].

Age	Percentage of Survival
75+ years	57.8%
20–44 years	91%
0–19 years	75.2%

**Table 4:** Molecular subgroups of ependymoma [41,51,52,53].

Anatomical/Molecular Classification		WHO Grade	Age Group
Supratentorial ependymoma (ST-)			
ST-SE	Subependymoma loss of chromosome 19	WHO-grade I	Adults
ST-EPN-YAP1	(Anaplastic) Ependymoma *YAP1*-fusion	WHO-grade II/III	Infants, Children
ST-EPN-RELA	(Anaplastic) Ependymoma Chromothripsis; *RELA*-fusion	WHO-grade II/III	Infants, Children, Adults
Posterior fossa ependymoma (PF-)			
PF-SE	Subependymoma loss of chromosome 19, 6p *TERT*-mutation	WHO-grade I	Adults
PF-EPN-A	(Anaplastic) Ependymoma Balanced genome	WHO-grade II/III	Infants, Children
PF-EPN-B	(Anaplastic) Ependymoma Chromosomal instability	WHO-grade II/III	Children, Adults
Spinal ependymoma (SP-)			
SP-SE	Subependymoma loss of chromosome 19, 6p	WHO-grade I	Adults
SP-MPE	Myxopapillary Ependymoma Chromosomal instability	WHO-grade I	Adults
SP-EPN	(Anaplastic) Ependymoma Chromosomal instability, *NF2* mutation	WHO-grade II/III	Adults
